# A case of acute cholecystitis with abnormally high CA19-9

**DOI:** 10.1186/s40792-023-01648-1

**Published:** 2023-05-03

**Authors:** Shinichiro Oinuma, Deguchi Yoshio, Syunsuke Omotaka, Takuya Terunuma, Shinya Kasagi, Hironobu Kimura, Noboru Yokoyama, Haruhiro Inoue

**Affiliations:** Digestive Disease Center, Showa University Koto Toyosu Hospital, 5-1-38 Toyosu, Koto-Ku, Tokyo, 135-8577 Japan

**Keywords:** Acute cholecystitis, CA19-9, Suspicious cancer

## Abstract

**Background:**

CA19-9 is strongly expressed in malignant tumors of the digestive system and is widely used as a marker for gastrointestinal cancer. In this report, we describe a case of acute cholecystitis in which CA19-9 was markedly elevated.

**Case presentation:**

A 53-year-old man was admitted to our hospital with a diagnosis of acute cholecystitis after being referred to our hospital with a chief complaint of fever and right hypochondrial pain. CA19-9 was abnormally high at 17,539.1 U/ml. Although the possibility of malignancy was considered, there was no obvious malignant lesion on imaging; the patient was diagnosed with cholecystitis, and laparoscopic cholecystectomy was performed the day after admission. The surgical specimen showed no malignant findings either grossly or in the final pathological examination. There were no complications in the patient’s postoperative course, and he was discharged from the hospital on the third postoperative day. CA19-9 level quickly returned to within normal range after surgery.

**Conclusions:**

In acute cholecystitis, CA19-9 levels exceeding 10,000 U/ml are very rare. We report a case of acute cholecystitis without malignant findings despite a high CA19-9 level.

## Background

CA19-9 is strongly expressed in malignant tumors of the digestive system and is widely used as a marker for gastrointestinal cancer. However, there are many reports of elevated levels of CA19-9 in cholecystitis, as well as in malignant diseases. When surgery is indicated, preoperative identification of malignancy is necessary. In the case of gallbladder malignancy, accurate preoperative information is essential to determine whether a change in the surgical technique needs to be considered. In the present study, we report a case of acute cholecystitis with markedly elevated CA19-9, and we discuss the clinical significance of CA19-9, including a review of the literature.

## Case presentation

**Fig. 1:**
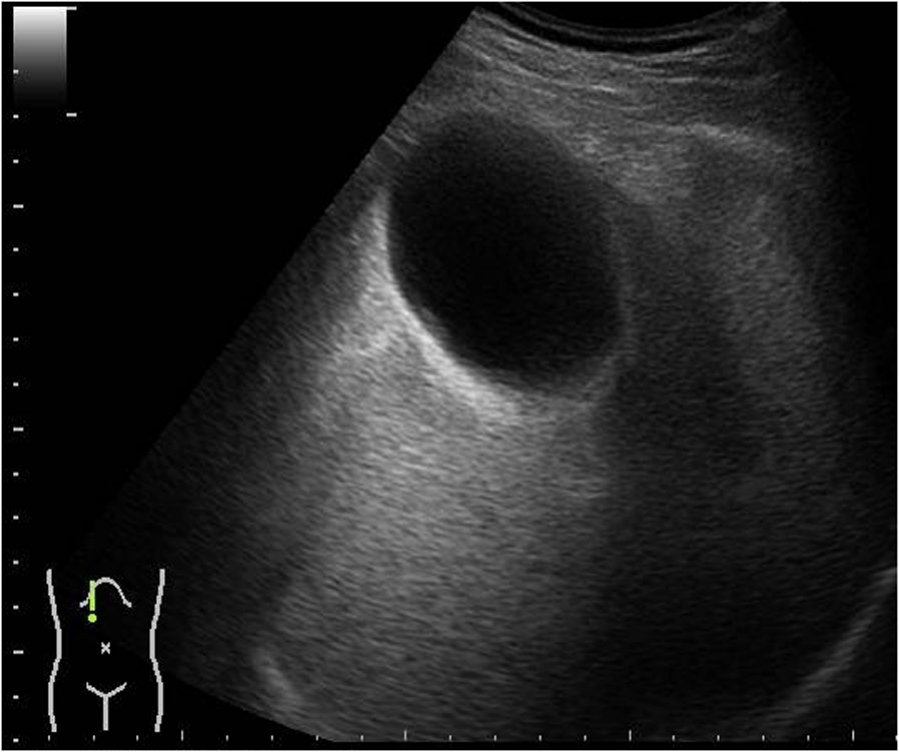
Ultrasound image of the gallbladder

**Fig. 2:**
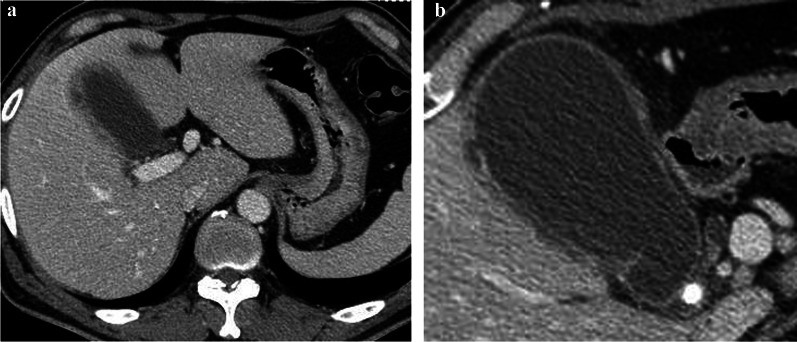
**a** CT showing thickening of the gallbladder wall. **b** Gallbladder stone impacted at the cystic duct

**Fig. 3:**
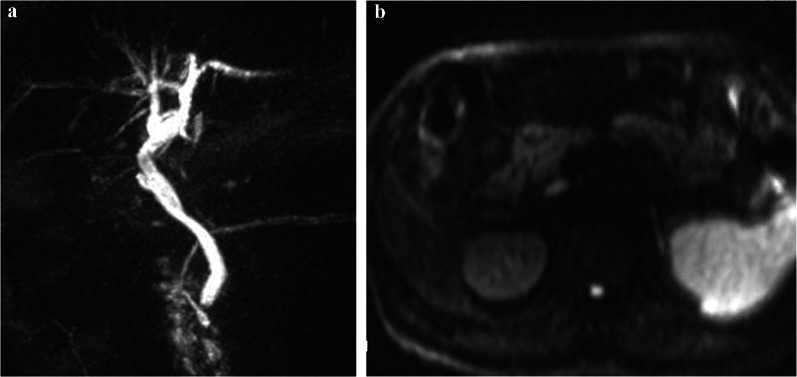
**a** Postoperative MRI image of the biliary tree. **b** No evidence of malignancy in the diffusion mode of postoperative MRI

**Fig. 4:**
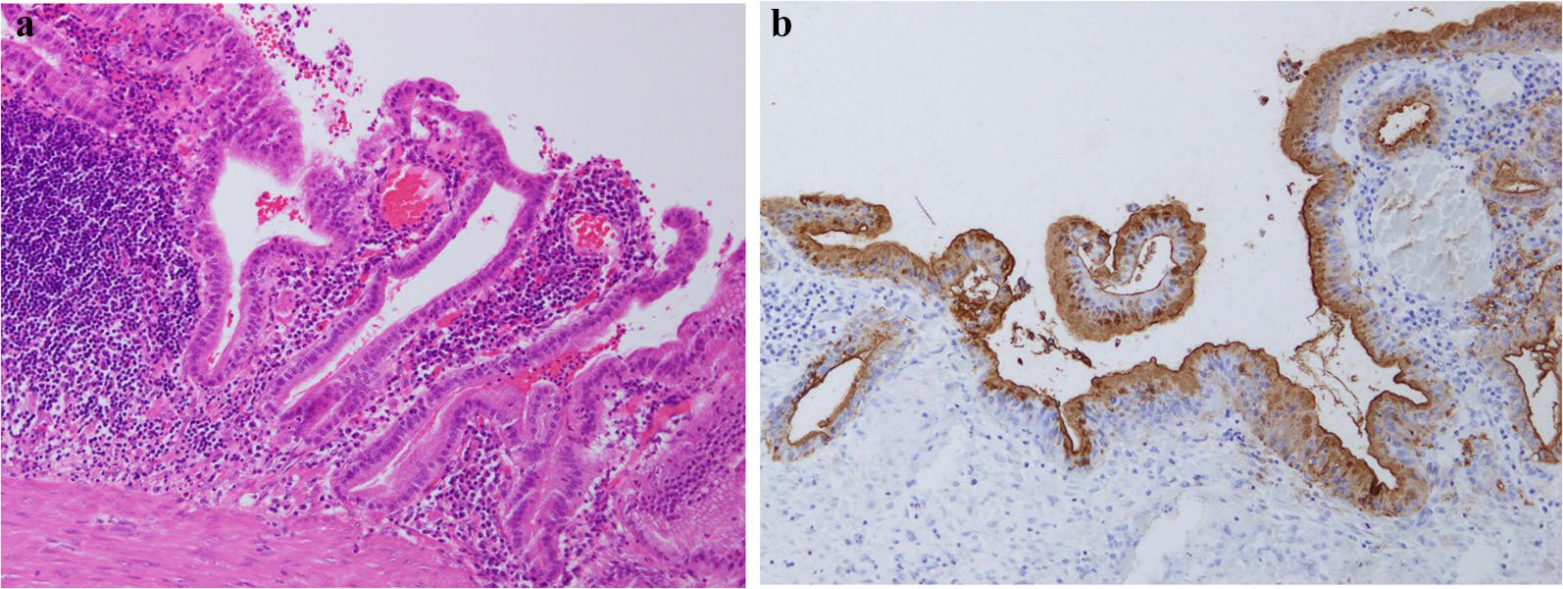
**a** Pathology showing a diffuse inflammatory cell infiltrate consisting mainly of neutrophils, lymphocytes, and plasma cells. **b** CA19-9 immunostaining is consistent with the findings of chronic cholecystitis

The patient was a 53-year-old male who had visited his previous physician 5 days before with fever and abdominal pain. He had a history of ileal resection for small bowel Crohn's disease 5 years prior. His body temperature was 35.6 °C, his blood pressure was 125/89 mmHg, and his pulse rate was 94/min. Conjunctival icterus was not noted. The patient had right hypochondrial pain and a positive Murphy's sign. Abdominal ultrasonography showed an enlarged gallbladder wall but no obvious intracholecystic calculus (Fig. 1). The laboratory data of the patient during hospitalization were as follows: white blood cell count of 4920/µl (normal range: 3500–9700/µl); C-reactive protein (CRP) level of 14.54 mg/dl (normal range: < 0.3 mg/dl); total bilirubin level of 2.8 mg/dl (normal range: 0.3–1.2 mg/dl); direct bilirubin level of 1.2 mg/dl (normal range: < 0.4 mg/dl); aspartate aminotransferase level of 27 U/l (normal range: 10–40 mg/dl); and alanine aminotransferase level of 30 U/l (normal range: 5–45 mg/dl). The serum carcinoembryonic antigen (CEA) level was 1.7 ng/ml (normal range: < 0.5 ng/ml), while the serum CA19-9 level was significantly elevated to 17,539.1 U/ml (normal range: < 37 U/ml). Contrast-enhanced CT showed gallbladder distention, wall thickening, gallstones in the gallbladder neck, and increased fatty tissue density around the gallbladder. The common bile duct and intrahepatic bile duct were not dilated, and there was no obvious neoplastic lesion (Fig. 2). Magnetic resonance cholangiopancreatography (MRCP) showed no anatomic disruption of the biliary system and no obvious neoplastic lesions (Fig. 3). Upper and lower gastrointestinal endoscopy revealed no specific findings and no obvious neoplastic lesions. During surgery, under general anesthesia and laparoscopic observation of the abdominal cavity, the omentum was observed to be attached to the gallbladder, and the gallbladder was enlarged and showed circumferential wall thickening. Since no obvious malignancy was found, the gallbladder was removed as planned. After removal, the specimen was immediately cut open for gross observation, but no obvious neoplastic lesions were found. The resected specimen showed impacted stones and several other small stones. Histopathological findings showed a diffuse inflammatory cell infiltrate consisting mainly of neutrophils, lymphocytes, and plasma cells and abscess formation on the gallbladder wall, which is a finding of chronic cholecystitis (Fig. 4). There were no complications in the patient’s postoperative course, and he was discharged on the fifth postoperative day. At the outpatient clinic in the second postoperative week, the patient’s CA19-9 level was within the normal range.

## Discussion

Sialyl Lewis A is a sugar chain detected in CA19-9, which is frequently used as a tumor marker. CA19-9 is increased in cancer, but it is also present in small amounts in noncancerous tissues and is often elevated in benign diseases. There are many epithelial cells in the biliary system that are thought to produce CA19-9. It is also present in the epithelial cells of all tubular tissues, including salivary glands, ovaries, kidneys, prostate, and the intestinal tract. Inflammatory stimulation at these sites is believed to cause CA19-9 to deviate from the intercellular space into the blood, resulting in high levels. In gastrointestinal malignant diseases, CA19-9 is useful for the diagnosis of pancreatic cancer [1]. CA19-9 has better diagnostic accuracy for pancreatic cancer than CEA, CA 125, DUPAN-2, TPA and PSTI/TATI [2]. CA19-9 has been found to be associated with high serum CA19-9 levels in benign diseases causing biliary obstruction [3]. Values greater than 10,000 U/ml in benign disease are very rare [4].

When CA19-9 is elevated with the presence of cholelithiasis or cholecystitis, ruling out malignancy is important in determining the course of treatment. In cases where diagnosis is difficult, other modalities such as endoscopic ultrasonography (EUS) may be considered for evaluation, including drainage and cytological diagnosis.

It has been reported that drainage has a positive effect on biliary tract obstruction and jaundice, resulting in a decrease in CA19-9 in all patients with benign disease [5].

In this case, there were no findings on imaging tests that clearly suggested gallbladder malignancy, and the patient underwent surgery, with improvement in CA19-9 observed in the postoperative course.

Tsen A and Giron F reported that the mechanisms of high CA19-9 in cholelithiasis and cholecystitis are as follows: ① inflammatory stimuli and elevated biliary pressure causing the bile duct epithelium to overproduce CA19-9; ② impaired outflow of the biliary ducts causing CA19-9 to escape into the interstitium and eventually into the blood; and ③ CA19-9, which then causes backflow and leakage of CA 19–9 into the systemic circulation [6, 7].

The improvement in CA19-9 with a reduction in inflammation and lifting of the origin of the obstruction suggests that the high CA19-9 was caused by overproduction of CA19-9 in the bile duct epithelium due to inflammatory stimuli and outflow obstruction, causing CA19-9 to migrate into the stroma and vascular plexus. CA19-9 immunostaining showed the gallbladder mucosal epithelial cytoplasm and inflammatory cells such as macrophages within the thickened gallbladder wall, which is consistent with the findings of chronic cholecystitis (Fig. 4).

We considered biliary drainage and conservative therapy to reduce jaundice, but the patient had typical acute cholecystitis requiring surgery due to gallstones in the gallbladder neck. The cause of the high CA19-9 level was judged to be inflammation, and surgery was performed.

The patient’s CA19-9 level returned to normal immediately after the surgery, and no malignant tumor was detected on pathological examination. The cause of the high CA19-9 was considered to be the result of deviation of CA19-9 from the epithelium due to cholelithiasis and cholecystitis. Since an improvement in CA19-9 was observed after a reduction in inflammation and release of the obstruction, both the origin of bile duct system obstruction and the presence of inflammation were considered the factors causing increased CA19-9.

Overinvasive surgery should be avoided if malignancy cannot be completely ruled out. The following treatment strategies can be considered: ① preoperative drainage, ② intraoperative rapid cytological diagnosis, and ③ observation of the progression of tumor markers over time. Bile cytology by drainage should be performed carefully when malignancy cannot be completely ruled out because of the risk of intra-abdominal seeding. If a facility has the capability to perform endoscopic drainage of the gallbladder through the transcholedochal duct, the procedure can be considered an option for preoperative examination. This is because even in the case of malignancy, endoscopic drainage will not disperse malignant cells into the abdominal cavity. Intraoperative rapid cytology is not a definitive diagnostic tool since the accuracy of the diagnosis depends on the size of the incision and the degree of inflammation.

Currently, laparoscopic cholecystectomy is the standard surgical technique for cholecystitis, but in principle, open cholecystectomy should be performed in patients with lymph node metastasis or other strong evidence of cancer [8]. In this case, there was no obvious wall thickening or tumor suspicious for cancer, the diagnosis of acute cholecystitis was made, and early surgery was performed according to the Tokyo guidelines [9].

Postoperatively, tumor markers showed rapid improvement, which may be useful in differentiating malignant tumors. In addition, if the cause of the high CA19-9 level cannot be determined, CT, including that for gynecological diseases, may be considered before additional invasive examinations; liver function, blood glucose, HbA1c, and additional thyroid function tests; or positron emission tomography (PET) scans performed in combination [10, 11].

CA19-9 levels exceeding 10,000 U/ml in acute cholecystitis are very rare, and this case description is a valuable report. By recognizing that CA19-9 can be elevated in benign disease without overlooking malignant disease, unnecessary invasive testing can be avoided, and the patient can be treated accordingly.

## Conclusions

Although there are many reports of cholecystitis with elevated CA19-9 levels, it is rare for such values to exceed 10,000 U/ml. This is an important issue in choosing the treatment strategy, including surgical procedures. More cases need to be accumulated in the future, but it should also be noted that even cases with very high CA19-9 levels may be benign.

## Data Availability

Data sharing is not applicable to this article, as no datasets were generated or analyzed during the current study.

## Abbreviations

MRCP: Magnetic resonance cholangiopancreatography
EUS: Endoscopic ultrasonography
PET: Positron emission tomography

## References

[1] Kim HJ, Kim MH, Myung SJ, Lim BC, Park ET, Yoo KS (1999). A new strategy for the application of CA19-9 in the differentiation of pancreaticobiliary cancer: analysis using a receiver operating characteristic curve. Am J Gastroenterol.

[2] Haglund C, Kuusela P, Roberts PJ (1989). Tumour markers in pancreatic cancer. Ann Chir Gynaecol.

[3] Shah N, Tetangco E, Arshad HMS, Raddawi H (2017). Mirrizi syndrome and markedly elevated levels of carbohydrate antigen 19–9 in the absence of malignant disease. Case Rep Gastrointest Med.

[4] Peterli R, Meyer-Wyss B, Herzog U, Tondelli P (1999). CA19-9 has no value as a tumor marker in obstructive jaundice. Schweiz Med Wochenschr.

[5] Mann DV, Edwards R, Ho S, Lau WY, Glazer G (2000). Elevated tumour marker CA19-9: clinical interpretation and influence of obstructive jaundice. Eur J Surg Oncol.

[6] Tsen A, Barbara M, Rosenkranz L (2018). Dilemma of elevated CA 19–9 in biliary pathology. Pancreatology.

[7] Giron F, Alcantar D (2020). Looks can be deceiving: a case report on the clinical value of CA 19–9 in obstructive jaundice. Cureus.

[8] Goetze TO, Paolucci V (2014). Immediate radical re-resection of incidental T1b gallbladder cancer and the problem of an adequate extent of resection (results of the German Registry "Incidental Gallbladder Cancer"). Zentralbl Chir.

[9] Takada T (2018). Tokyo guidelines 2018: updated Tokyo guidelines for the management of acute cholangitis/acute cholecystitis. J Hepatobiliary Pancreat Sci.

[10] Kim S, Park BK, Seo JH, Choi J, Choi JW, Lee CK (2020). Carbohydrate antigen 19–9 elevation without evidence of malignant or pancreatobiliary diseases. Sci Rep.

[11] Akimoto S, Banshodani M, Nishihara M, Nambu J, Kawaguchi Y, Shimamoto F (2016). Acute cholecystitis with significantly elevated levels of serum carbohydrate antigen 19–9. Case Rep Gastroenterol.

